# Imported spring onions related to the first recorded outbreak of enteroinvasive *Escherichia coli* in Denmark, November to December 2021

**DOI:** 10.2807/1560-7917.ES.2023.28.15.2200572

**Published:** 2023-04-13

**Authors:** Mia Torpdahl, Emily Dibba White, Susanne Schjørring, Malene Søby, Jørgen Engberg, Anne Line Engsbro, Hanne M Holt, Lars Lemming, Lisbeth Lützen, Bente Olesen, John E Coia, Charlotte Kjelsø, Luise Müller

**Affiliations:** 1 Statens Serum Institut, Copenhagen, Denmark; 2 Danish Veterinary and Food Administration, Glostrup, Denmark; 3 Department of Clinical Microbiology, Zealand University Hospital, Slagelse, Denmark; 4 Department of Clinical Microbiology, Copenhagen University Hospital, Hvidovre, Denmark; 5 Department of Clinical Microbiology, Odense University Hospital, Odense, Denmark; 6 Department of Clinical Microbiology, Aarhus University Hospital, Aarhus, Denmark; 7 Department of Clinical Microbiology, Lillebaelt Hospital, Vejle, Denmark; 8 Department of Clinical Microbiology, Copenhagen University Hospital, Herlev and Gentofte, Denmark; 9 Department of Clinical Diagnostics, Hospital South West Jutland, Esbjerg, Denmark

**Keywords:** Enteroinvasive *Escherichia coli*, EIEC, *Shigella*, ipaH, outbreak, imported vegetables, spring onions, ready-to-eat products

## Abstract

Between November and December 2021, the first ever recorded outbreak of enteroinvasive *Escherichia coli* in Denmark occurred at national scale. We describe the investigation of this outbreak, which was initially recognised in early December 2021. A total of 88 cases (58 female; 30 male) with a median age of 52 years (range: 0–91) were detected by PCR-based diagnostic methods. Case ascertainment was complicated by current culture-free diagnostic procedures, with only 34 cases confirmed by culture, serotyping and whole genome sequencing. Isolates from cases grouped into two serotypes (O136:H7 and O96:H19), which was supported by whole-genome-sequence-phylogeny, also yielding two clusters. Interviews of 42 cases and traceback investigation pointed towards consumption of ready-to-eat salads as the outbreak cause. While the ready-to-eat salads comprised different vegetables, imported spring onions were the only common ingredient and thus the likely source. Environmental investigations failed to recover outbreak strains. This report highlights the value of fast typing (here O-typing) to confirm cases in an outbreak situation. Timely communication and data sharing are also important, and were facilitated by the national collaboration between relevant laboratories, the public health institute and the veterinary and food administration. High hygiene standards for imported fresh vegetables intended for ready-to-eat products are essential.

**Key public health message**

**What did you want to address in this study?**

We wanted to control a large national outbreak in Denmark caused by enteroinvasive *Escherichia coli* (EIEC), a food-borne bacterial pathogen giving diarrhoeal disease. To know the extent of the outbreak, we first wanted to identify outbreak cases, that is, individuals sharing the particular EIEC strain involved in the outbreak. This would allow interviewing cases to discover how they got infected, which in turn might help us uncover the source of the outbreak to stop it.

**What have we learnt from this study?**

Based on PCR diagnostic methods, 88 people were possible outbreak cases. As PCRs in use did not distinguish EIEC from *Shigella*, another pathogen giving similar symptoms, confirming possible cases as outbreak cases required bacterial culture, then O-typing or genome sequencing. Thirty-four cases were confirmed. For this O-typing was faster than genome sequencing. Interviews and traceback investigations revealed imported spring onions in ready-made salads as the outbreak source.

**What are the implications of your findings for public health?**

Fast typing methods are important for confirming cases during an epidemic. During a food-borne outbreak, surveillance needs to be rapidly adapted, and here close collaboration between clinical microbiology laboratories, the public health institute and the veterinary and food administration was very valuable. The outbreak underlines that high hygiene standards for imported fresh vegetables should be maintained when these are to be used in ready-to-eat products.

## Background

Enteroinvasive *Escherichia coli* (EIEC) is a Gram-negative bacteria causing diarrhoeal disease. EIEC is transmitted via the faecal–oral route, with a usual incubation period of 1–3 days; infections are frequently related to contaminated food and water [1,2]. In Denmark, disease caused by EIEC is mostly observed in returning travellers, but secondary transmission from person-to-person may occur [3]. In Europe, outbreaks of EIEC in 2012, 2014 and 2017 have been reported and, for all of these, contaminated vegetables were suspected as the source [4-6].

Clinically, EIEC infections present either with watery diarrhoea or dysentery. EIEC invade the epithelial cells of the large intestine in the same manner as *Shigella* and symptoms resulting from EIEC infection are clinically indistinguishable from shigellosis [7]. Studies have shown that *E. coli* and *Shigella* species have high genomic and phenotypic similarity, leading to propose that *Shigella* species should be reclassified as a subspecies of *E. coli* [8,9].

Diagnostics of EIEC in Denmark are done locally through 10 different clinical microbiology laboratories situated at hospitals in the five Danish regions. The criteria for carrying out an EIEC diagnostic vary. Some laboratories test all faecal samples for diarrhoeagenic *E. coli* including EIEC, while others test only faecal samples from suspected patients, based on their age, travel history and presence of bloody diarrhoea. The PCR diagnostic assays target the invasive plasmid gene (*IpaH*) shared by both *Shigella* spp. and EIEC [10]. Culture is required to differentiate the two species, and if culture is not possible or unsuccessful, faecal specimens are considered positive for the combination *Shigella*/EIEC. Detection of *Shigella*/EIEC is voluntarily notified as part of the Danish laboratory surveillance, where episodes are irregularly reported by the clinical microbiology laboratories to Statens Serum Institut (SSI), the national public health institute. All isolates from successfully cultured samples are furthermore routinely sent on a voluntary basis to SSI for further characterisation.

### Outbreak detection

On 10 December 2021, the clinical microbiology laboratory at Slagelse hospital in Region Zealand reported observing an increase between 5 and 8 December of patients diagnosed with domestically-acquired EIEC. During this period, this laboratory had detected a total of five such patients. On the same day (10 December), SSI identified one EIEC isolate with serotype O96:H- and three with serotype O136:H7 (two of which originated from Slagelse hospital), the latter a type never found in human samples from Denmark before. In total, between 6 and 10 December, SSI had received six EIEC isolates from three different regions of Denmark, exceeding the total number of EIEC isolates that were received in the months of December of the 2 previous years. A national outbreak was therefore declared on 13 December.

## Methods

### Diagnostics of enteroinvasive *Escherichia coli*

All local clinical microbiological laboratories in Denmark perform PCR for the invasive plasmid gene (*IpaH*), shared by both *Shigella* spp. and EIEC, when a patient needs to be tested (according to their laboratory testing criteria). The PCR is done either directly on faecal samples or after a culture step on SSI Enteric Medium (SSI Diagnostica, Hillerød, Denmark). PCR assays are commercial or in-house developed assays [11-15] and faecal samples positive for the *IpaH* gene are cultured on SSI Enteric Medium followed by a second PCR or more PCR steps to try and identify a single colony positive for the *IpaH* gene. Identification of the bacterial species is performed by matrix-assisted laser desorption-ionization time-of-flight (MALDI-TOF; Bruker, Copenhagen, Denmark), biochemical tests including motility, indole test, glucose/gas and negative agglutination with *Shigella* specific antisera (Sifin Diagnostics GmbH, Berlin, Germany). Antimicrobial susceptibility testing is performed using disk diffusion [16]. During the outbreak, active case ascertainment was deployed and all laboratories were requested to report PCR results and send isolates (if available) in real-time to SSI.

### Case definition

Based on the microbiological data, epidemiological descriptive analysis and the interviews of patients who pointed towards the same common source, a confirmed case was defined as an individual with a culture-positive EIEC, with O136:H- or O96:H19 conventional serotypes and/or O136:H7, ST270#1 or EIEC O96:H19, ST99#1 based on sequence data (see next sections), with onset of illness from 15 November to 31 December 2021. A possible case was defined as an individual with a PCR-positive for *Shigella*/EIEC. Cases were excluded if (i) isolates were different from O136:H- or O96:H19 when serotyped, (ii) they reported travels within 14 days before disease onset and isolate serotype was unknown.

### Microbiological and molecular typing

Upon reception at SSI, isolates were immediately verified as part of the outbreak using *E. coli* serotyping with antisera against *E. coli* O antigens and *E. coli* H antigens [17,18].

Single colonies were used to extract genomic DNA with an enzymatic pre-lysis step before automated purification using the MagNA Pure 96 DNA and Viral NA Small Volume Kit and DNA Blood ds SV 2.0 protocol (Roche Diagnostics, Hvidovre, Denmark). Libraries were constructed, and sequencing was performed using the Nextera XT Kit (Illumina, Little Chesterford, United Kingdom (UK)) and 300-cycle kits on the NextSeq 550 (Illumina) platform according to the manufacturer’s instructions. Quality control of the sequencing data was done using bifrost (https://github.com/ssi-dk/bifrost), in which isolate sequences were re-sequenced in case of contamination with > 5% of another genus, as well as sequences representing *E. coli* isolates with genome sizes outside the range of 4.6–5.6 Mbp. Sequences were used for in-silico serotyping [19] and detection of antimicrobial resistance genes [20].

Sequences were further analysed using the Enterobase core genome multilocus sequence typing (cgMLST) scheme [21,22], in BioNumerics (BioMérieux, Belgium). A genetic related cluster was defined by the population structure in the relevant sequence type (ST), usually within a few allelic differences applying single linkage. Whole genome sequencing (WGS) was used to assign cluster identification numbers to the cases included in the outbreak as previously described for *Campylobacter* [23]. Two representative outbreak sequences from Denmark were deposited to the European Nt Archive (https://www.ebi.ac.uk/ena; accession numbers ERR7916769 (2112T45202) and ERR7916767 (2112S29924)).

### Antimicrobial susceptibility testing at Statens Serum Institut

Four selected isolates were tested for antimicrobial susceptibility by broth microdilution with the EUVSEC3 panel using the Sensititre Automated Microbiology System (Termo Fischer Scientific, Roskilde, Denmark) in accordance with recommendations from the European Centre for Disease Prevention and Control (ECDC) [24] and using European Committee on Antimicrobial Susceptibility Testing (EUCAST) breakpoints [16].

### Epidemiological investigations

As it was the first time that an EIEC outbreak occurred in Denmark, there was no standard questionnaire available for this pathogen, so a trawling questionnaire usually used for Shiga toxin-producing *E. coli* (STEC) and *Salmonella* outbreaks was employed. Cases were interviewed by telephone using systematic hypothesis-generating questionnaires about disease onset, symptoms, grocery shops and food consumption 7 days before disease onset. The questionnaire included several food items e.g. vegetables, fruits, fresh- and dried spices, berries, nuts and snacks. Since food consumption can be hard to recall, four options were presented; ‘Yes’/’Probably yes’/’No’/’Probably not’. In the analyses, we combined ‘Yes’ and ‘Probably yes’ as reported intake and ‘No’ and ‘Probably not’ as not reported intake. Attempts were made to interview all possible and confirmed cases. Cases were interviewed as soon as they were reported. The first 17 cases were interviewed in December 2021 and asked about all the items in the questionnaire. As our suspicion grew towards ready-to-eat salad with fresh cabbage, a question on the consumption of such a type of food was inserted in the questionnaire, creating a possibility to shorten it. If the interviewed case reported intake of ready-to-eat fresh cabbage salads in the 7 days before disease onset, the interview would then end. If the interviewed case did not report intake of ready-to-eat fresh cabbage salads in the 7 days before disease onset, the rest of the questionnaire would then be completed.

### Traceback and environmental investigations

A traceback investigation was conducted to determine the common ingredient in the different ready-to-eat salads with fresh cabbage that the cases had bought. Cases were asked to send the name of the grocery-shop, date of the grocery-shopping and the credit-card-number used. The Danish Veterinary and Food Administration (DVFA) found that the common ingredient in the different types of ready-to-eat salads was spring onions imported pre-processed from the Netherlands and included by the Danish producer in the ready-to-eat salads.

On 22 December, DVFA retrieved from the producer of ready-to eat-salads, a total of 20 samples. These samples included 10 samples from spring onions (originating from two different batches of spring onions, whereby five samples were taken from each batch), five samples of ready-to-eat salads with pointed head cabbage and five samples of salad with red pointed head cabbage (both salads containing spring onions). The samples were analysed for *Shigella* spp., EIEC, and STEC. The method “presumptive *Shigella* spp. detection in foods” (NMKL method Number 151, 2019) from the Nordic Committee on Food Analysis (https://www.nmkl.org/) was used for *Shigella* spp., while the RealStar EHEC PCR Kit 2.0 (altona Diagnostics GmbH, Hamburg, Germany) was used for both EIEC and STEC. The producer of ready-to-eat salads took five samples in each of two batches of spring onions for investigation between 27 December and 7 January.

DVFA further created a news notification on 23 December 2021 in the Rapid Alert System for Food and Feed (RASFF) (reference number: 2021.7145), describing the outbreak. Moreover, the authorities in the Netherlands were asked to follow up on the notification and share the results of their investigations. On 28 January 2022, the Dutch authorities informed that a microbiological study had been carried out in one batch of spring onions, sampled on 19 November 2021 at the pre-processing Dutch site. 

### International communication about the outbreak

In addition to DVFA posting a notification in RASFF, information on the episodes and isolates’ characteristics were included in an event report that was posted by SSI on the ECDC Epidemic Intelligence Information System (EPIS) (2021-FWD-00104) for other European countries to report cases if they detected any.

## Results

### Phenotypic and whole genome sequence analyses

All isolates of EIEC submitted to SSI were characterised using conventional serotyping for a fast identification of cases who should be considered for interview. In total, 34 isolates were serotyped, 24 were serotyped as O136:H- and 10 were serotyped as O96:H19. Two isolates of each serotype were tested for antimicrobial susceptibility to 16 antibiotics. They were all fully susceptible to all antibiotics tested and this was further confirmed from the WGS data. WGS was also used for in-silico serotyping as well as sequence typing and O136:H7 with ST270, as well as O96:H19 with ST99, were detected. The sequence-based cluster analysis revealed two separate clusters (data not shown) and within each cluster there were no more than three allelic differences between sequences. The clusters were given the ID ST270#1 and ST99#1, reflecting that these were the first identified clusters within the two ST-groups as well as in general in EIEC isolated in Denmark.

### Surveillance data

**Figure 1:**
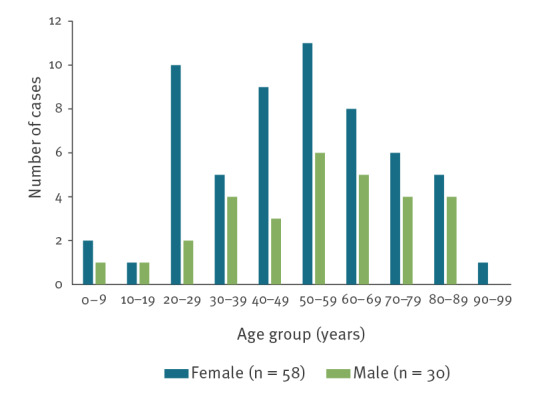
Age and sex distribution of cases of an EIEC outbreak in Denmark, November–December 2021 (n = 88)

After the outbreak was declared on 13 December, cases reported as *Shigella*/EIEC were added to a line list on a daily basis together with cases linked to a culture-positive EIEC. Seven of the 10 Danish microbiological laboratories reported episodes and/or sent isolates, which were part of the outbreak. A total of 88 cases were detected including 34 confirmed and 54 possible. Overall the cases were aged 0–91 years old, with a median age of 52 years; 58 cases (66%) were female and 30 (34%) male (Figure 1). Cases resided in four of five Danish regions; the region without cases being Region Northern Jutland. Symptoms were known for 41 cases, 40 had diarrhoea and 10 cases had bloody diarrhoea. Twenty-six cases were hospitalised and three cases died within 30 days after having tested positive for EIEC.

EIEC: enteroinvasive *Escherichia coli.*

**Figure 2:**
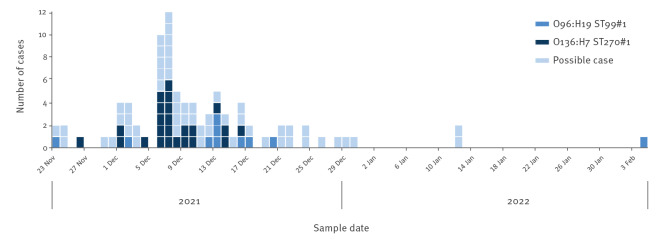
Date of sampling of cases of an EIEC outbreak stratified according to serotype, Denmark, November 2021–February 2022 (n = 88)

Date of symptom onset was known for 40 cases and ranged from 15 November to 24 December 2021. Date of sampling ranged from 23 November to 4 February 2022 and peaked in week 49 (6–13 December) (Figure 2). No other cases were reported from other European countries, based on the replies to the event posted on the ECDC EPIS.

EIEC: enteroinvasive *Escherichia coli.*

Three cases have sample dates in January/February 2022, but their onset of illness was in November/December 2021.

### Epidemiological investigation

In total, 42 of 88 cases were interviewed. The serotype-distribution among the interviewed were 14 cases with O136:H7, 10 cases with 096:H19 and 18 cases with *Shigella*/EIEC. During interviews, the majority of cases who answered the specific questions had reported consumption of vegetables such as cucumbers (28/38), tomatoes (23/38), apples (17/24), carrots (23/38), green salad (23/38) and ready-to-eat fresh cabbage salad (27/41) before disease onset. In total, 27 of 42 cases reported consumption of ready-to-eat salad with fresh cabbage and 20 of 27 used the same grocery chain. Among those who reported intake of ready-to-eat fresh cabbage salad, several types were mentioned, e.g. coleslaw, scallion salad and winter salad. Interviews showed the same consumption pattern for all cases regardless of the two different serotypes (O136 and O96), supporting that both serotypes should be included in the outbreak.

### Traceback investigation

A credit card investigation showed that seven of eight cases had bought different ready-to-eat fresh cabbage salads in the period of 15 November to 3 December 2021. Five of the seven ready-to-eat salads from the credit card investigation were known to be produced by the same Danish producer. The two remaining salads were suspected to be also produced by this company. Spring onions were the only common ingredient in the implicated salads. The spring onions originated from Egypt and were delivered from a Dutch food business operator who prior rinsed and root cut the product. No specific batches of spring onions were identified as being the source of the outbreak.

### Environmental investigations

DVFA examined samples of both spring onions and ready-to-eat salads, on 22 December. *Shigella* and/or EIEC were not detected by means of culturing or PCR. On 19 November 2021 the Dutch supplier carried out an analysis of a batch of spring onions. Furthermore, the producer of ready-to-eat salads examined samples of two batches of spring onions between 27 December and 7 January. No *E. coli* were detected in any of the spring onions analysed.

### Outbreak control measures

#### National communication

Information on the outbreak was published on the SSI outbreak disease page (https://www.ssi.dk/sygdomme-beredskab-og-forskning/sygdomsudbrud) on 22 December 2022 At that stage, this posting only mentioned ready-to-eat cabbage salads as the probable outbreak cause, with no accompanying news story. When spring onions were discovered as the common salad ingredient, however, and suspicion grew towards them, a news story was released on 27 December. On the same day several media picked up the story. No recall of food was initiated since the imported spring onions were part of ready-to-eat fresh salads, which have a short shelf life. Further, as the salad producer had changed its spring onion supplier, and the numbers of incoming cases were declining at that time, we did not expect the outbreak to continue.

#### International communication and further actions

Upon request, the Dutch authorities informed DVFA that the deliveries of spring onions had only been directed to the one recipient in Denmark identified through the outbreak investigation. At the time of the investigation, the suspected spring onions were no longer on the market. Moreover, in samples of subsequent batches of spring onions EIEC was not detected. Hence, only a RASFF notification in the form of a ‘RASFF News’ was initiated to inform other European Union countries about the outbreak and the spring onions as its suspected source. No withdrawal of foods was initiated, neither in Denmark nor in other European countries. We are not aware of any food-contaminated products found in Europe which could be related to this outbreak. However, in the same period of time the UK had an outbreak with *Shigella sonnei* where spring onions originating from the same producer in Egypt were the suspected source [25].

The International Food Safety Authorities Network (INFOSAN) Secretariat informed DVFA on 25 January 2022 that they had contacted the INFOSAN Emergency Contact Point in Egypt concerning the outbreak in Denmark, based on the news notification to RASSF. The INFOSAN Secretariat did not receive any response from the contact point in Egypt. On the other hand, the Danish embassy in Egypt contacted the DVFA regarding the outbreak on 21 February 2022, offering to assist with the investigations. On 15 March 2022, DVFA asked it for any clues as to why spring onions could have been contaminated. The embassy returned on 23 May 2022 with information of reports from 2021 indicating several instances of the Nile overflowing its banks. The flooding had peaked with the onset of the rainy season in May 2021 and continued until December that same year. The embassy informed that, as a general rule, the Nile flood in Egypt occurs in the 3 months of August, September and October. However, in 2021 the floods periodically were above average extending the flooding period.

## Discussion

We identified an outbreak of EIEC linked to consumption of ready-to-eat fresh salads with imported spring onions as the only common ingredient. The outbreak highlights the importance of timely reporting EIEC cases as well as of culturing and characterising isolates to distinguish outbreak cases from sporadic ones.

SSI is responsible for the national surveillance of EIEC and highly relies on reporting of *Shigella*/EIEC by the clinical microbiology laboratories. A delay in reporting reduces the ability of case-based surveillance to timely detect outbreaks. Moreover, because laboratory reporting of cases depends on their finding, the testing of people needs to be sufficient. EIEC may be under-reported by the clinical microbiology laboratories for two reasons. First because bacterial culture followed by PCR or direct PCR on faecal samples is needed for detection, and culture can be a challenging technique. Second, not all relevant faecal samples may be tested as some laboratories test only faecal samples from suspected patients, based on their age, travel history and presence of bloody diarrhoea.

To identify outbreak cases, culture and subsequent confirmation of the bacterial species, as well as characterisation and susceptibility testing of isolates are very important. In investigations where many of the PCR-positive samples are not subsequently successfully cultured, speciated or typed, a less specific case definition has to be applied, which can lead to inclusion of sporadic cases not related to the outbreak. This complicates the investigation and the possibility to demonstrate a common source.

Nevertheless, in the present outbreak, interviews with possible cases and confirmed cases, regardless of serotypes, pointed towards ready-to-eat salads as the cause. This cause seemed plausible because the outbreak occurred when travel-related cases were scarce. Indeed, due to travel restrictions from March 2020, imposed under the COVID-19 pandemic, the level of food-borne bacterial infections had decreased in the country in 2020 compared to 2019, especially for individuals with a travel history before disease onset [26]. EIEC is mostly isolated from such individuals in Denmark, and from 2019 to 2020 the total number of people diagnosed with EIEC had decreased by 69% (data not shown). The high number of cases confirmed with EIEC from early December 2021 suggested an outbreak of EIEC due to a source present nationally.

Serotyping was sufficient to identify outbreak cases as both outbreak serotypes were very rare in Denmark. Because this method (O-typing) took less time than WGS (1–2 days vs 7–10 days respectively), it was deemed superior for rapid identification of cases. This allowed to start interviews within a few days. Time is essential when identifying and interviewing food-borne outbreak cases to link an outbreak to the consumption of a specific food and to implement relevant control measures to stop it.

There are several limitations to this study. There may have been undetected cases in Denmark as well as Europe due to the under-reporting or sometimes even lack of reporting of EIEC. Further, culture-independent diagnostic tests (CIDT)s currently being used in the diagnostic laboratories made separating outbreak and sporadic cases difficult. We used a trawling questionnaire usually used for STEC and *Salmonella* outbreaks, because an EIEC outbreak had not been previously experienced in Denmark, so we did not have a standard questionnaire for this pathogen. Finally, although the epidemiological and traceback investigations pointed to ready-to-eat salads with spring onions as an ingredient, we did not succeed in detecting the outbreak strains in any of the samples tested.

### Conclusions

The current study emphasises the relevance of timeliness in detecting *Shigella*/EIEC outbreaks, reporting cases and typing available isolates. This increases the possibility of elucidating the cause through case interviews. This report also highlights the importance of a good collaboration at country-level between the different bodies conducting surveillance, in this instance, the clinical microbiology laboratories, SSI and the DVFA. Communicating diagnostic procedures and signals identified locally at national and international level is also important.
